# Review of Sensor Technology to Support Automated Air-to-Air Refueling of a Probe Configured Uncrewed Aircraft

**DOI:** 10.3390/s23020995

**Published:** 2023-01-15

**Authors:** Jonathon Parry, Sarah Hubbard

**Affiliations:** 1 Polytechnic Institute, Purdue University, West Lafayette, IN 47907, USA; 2 School of Aviation and Transportation Technology, Purdue University, West Lafayette, IN 47907, USA

**Keywords:** automated air-to-air refueling, A3R, computer vision, autonomous aerial refueling, AAR, UA, RPA, UAV, UAS

## Abstract

As technologies advance and applications for uncrewed aircraft increase, the capability to conduct automated air-to-air refueling becomes increasingly important. This paper provides a review of required sensors to enable automated air-to-air refueling for an uncrewed aircraft, as well as a review of published research on the topic. Automated air-to-air refueling of uncrewed aircraft eliminates the need for ground infrastructure for intermediate refueling, as well as the need for on-site personnel. Automated air-to-air refueling potentially supports civilian applications such as weather monitoring, surveillance for wildfires, search and rescue, and emergency response, especially when airfields are not available due to natural disasters. For military applications, to enable the Air Wing of the Future to strike at the ranges required for the mission, both crewed and uncrewed aircraft must be capable of air-to-air refueling. To cover the sensors required to complete automated air-to-air refueling, a brief history of air-to-air refueling is presented, followed by a concept of employment for uncrewed aircraft refueling, and finally, a review of the sensors required to complete the different phases of automated air-to-air refueling. To complete uncrewed aircraft refueling, the uncrewed receiver aircraft must have the sensors required to establish communication, determine relative position, decrease separation to astern position, transition to computer vision, position keep during refueling, and separate from the tanker aircraft upon completion of refueling. This paper provides a review of the twelve sensors that would enable the uncrewed aircraft to complete the seven tasks required for automated air-to-air refueling.

## 1. Introduction

The concept of air-to-air or aerial refueling has existed for over a century and it provides significant benefits by increasing the range and flight time of the aircraft [1]. A series of refueling engagements increases the range and flight time of an aircraft to such a point that human limitations become the limiting factor of the aircraft, as prescribed by the Federal Airway Administration (FAA) [2] or the applicable service instruction. With the use of remotely piloted aircraft (RPA) or uncrewed aircraft (UA), crew limitations no longer exist, and aerial refueling can dramatically extend mission capabilities for military use, as well as potential applications for civilian use [3].

Given the strategic advantage aerial refueling provides, aerial refueling has been used for military operations for decades. Due to the cost of integration of aerial refueling systems, the requirement for close formation flying, and the safety risks associated with the task, aerial refueling has predominately been limited to military operations only. During operations in support of Desert Storm, the United States Air Force (USAF) flew nearly 15,000 sorties dedicated to aerial refueling in which their fleet refueled approximately 46,000 aircraft with approximately 700 million pounds of fuel [4]. The requirement to refuel will only increase in future conflicts as standoff ranges for tankers increase due to kinematic capabilities of the weapon systems defending a hostile strategic location.

Studies have also been accomplished to examine the potential benefit of conducting aerial refueling for civilian airline operations. A study conducted in 2006 showed that civilian aircraft could see fuel savings of approximately 30–40% while being able to employ smaller, more efficient aircraft to complete longer range missions [3]. Secondary benefits of this include decreasing the size of runways required for aircraft that could aerial refuel, which would drastically increase the flexibility of airlines to provide services to smaller airports [5]. An increase in aviation infrastructure and additional training for pilots would be required to create, train, and manage a civilian tanker fleet for airline operations, similar to the recommended military qualification program provided by the North Atlantic Treaty Organization (NATO) [6]. It would also be necessary to change current aircraft separation requirements while reassessing and accepting the risks of having an aircraft carrying hundreds of people fly in close formation with another aircraft. Policies for fuel requirements that would facilitate travel to the nearest divert in the event of an unsuccessful engagement or “sour tanker” (a tanker unable to transfer fuel) would need to be developed as well. Finally, understanding the likelihood of and severity of cascading delays throughout a global network of flights when an airline cannot complete aerial refueling would need to be accounted for in a resilient airline scheduling program [7].

Advancing technologies provide promise for the use of automated air-to-air refueling (A3R). As technology advances, A3R is expected to overcome the traditional limitations of aerial refueling associated with aircraft handling and pilot skills. A3R is synergistic with the increasing use of UA or RPA, and A3R increases the capabilities for military applications, as well as the potential for future civilian applications with small Unmanned Aircraft Systems (sUAS). For nomenclature, the term UA (used by the United States Navy (USN)) will be used for the remainder of this paper and will encompass UA, RPA (used by the USAF), UAS, and sUAS (used in Part 107 operations [8]).

The USN published their vision for Naval Aviation in the 2030–2035 timeframe and a heavy emphasis was placed on the importance of Crewed UnCrewed Teaming (CUC-T) [9]. In the near future, the USN looks to deploy their first aircraft carrier-based UA to support tanking and information, surveillance, reconnaissance, and targeting (ISR-T) with the MQ-25 Stingray [9]. More information on the MQ-25 will be provided in Section 2.4, The importance of a UA capable of providing fuel airborne to extend all Carrier Air Wing (CVW) crewed platforms cannot be understated. Furthermore, the vision also outlines that as autonomy and learning enabled systems (LES) mature in capabilities, the USN will continue to evaluate the portions of crewed and uncrewed aircraft to ensure CVW lethality.

A3R supports a wide variety of military applications, including surveillance, strategic engagement, large scale, and complex operations. A3R could also support a wide variety of civilian operations UAs currently support to include weather monitoring [10], surveillance for wildfires [11], search and rescue [12], and emergency response and disaster management [13]. For both military and civilian operations, A3R reduces the need for ground-based infrastructure, such as intermediate refueling airfields, and provides additional flexibility for extended flights. A3R can provide significant advantages for large aircraft, as well as for small aircraft, including UAS. Without A3R, small uncrewed aerial vehicles have a limited range due to fuel constraints imposed by their small size and limited load capacity. Use of A3R can provide strategic and tactical advantages both for individual aircraft and for cluster applications [14].

This paper provides a brief history of air-to-air refueling, presents the framework for probe-and-drogue refueling, discusses innovations in automated air-to-air refueling, describes the tasks required to complete air-to-air refueling, and presents a review of the sensor technologies to support A3R for a probe configured UA. The probe-and-drogue method of air-to-air refueling is the focus of this paper and is used by the USN, as well as other NATO combat aircraft.

## 2. Background

### 2.1. Air-to-Air Refueling—Brief History

On 17 December 1903, Orville and Wilbur Wright executed the first controlled flight in a heavier-than-air aircraft under power [15]. Five months prior to the 20th anniversary of that historic day, another first was accomplished in the world of aviation. On 27 June 1923, over Rockwell Field in San Diego, Lt. Virgil Hine and 1st Lt. Frank Seifert flew a DH-4B to serve as a tanker aircraft for Capt. Lowell Smith and Lt. John Richter in their DH-4B to complete the first recorded instance of air-to-air refueling [1]. Using a fifty-foot rubber hose trailing from the tanker, the receiver aircraft grabbed the hose to complete the refueling connection for the second DH-4B to receive gas. Until the late 1950′s, all United States combat aircraft used a method of air-to-air refueling known as “hose-and-drogue”, also commonly referred to as probe-and-drogue. In 1950, Boeing demonstrated a boom-equipped tanker in response to challenges controlling the hose in bad weather and to support advancing and faster aircraft, which the US Air Force (USAF) pursued for future platform [16]. Today, NATO combat aircraft use a mix of the probe-and-drogue and boom-equipped tanker methods. The USN fixed wing aircraft, such as the F/A-18 E/F or F-35 B/C, rely on the probe-and-drogue method to receive fuel from drogue configured aircraft, including the KC-130 or Air Refueling Store (ARS) equipped F/A-18E/F. The USAF fixed wing aircraft, including the F-15E and F-22A, rely on boom-equipped tankers, such as the KC-10, KC-135, and the new KC-46, to complete air-to-air refueling [6].

**Figure 1:**
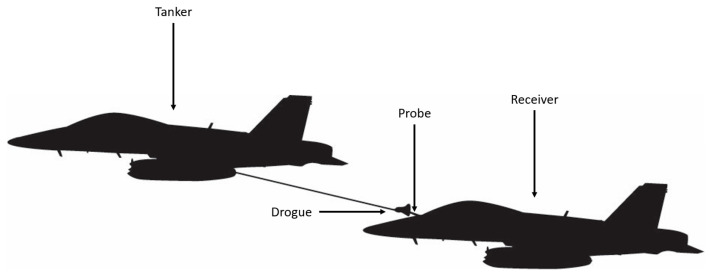
Example of probe and drogue refueling.

The primary difference between probe-and-drogue refueling and boom-equipped refueling derives from the aircraft completing the engagement. During a probe-and-drogue configured engagement, the pilot in the receiving aircraft must guide the probe tip into the coupler of the drogue to complete the engagement (Figure 1).

**Figure 2:**
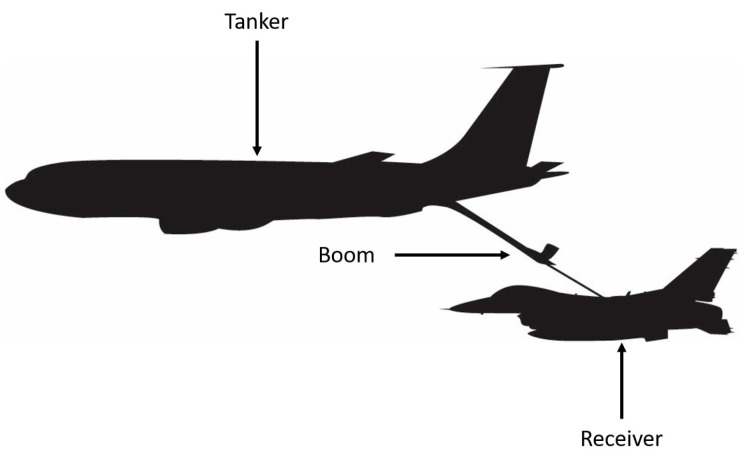
Example of boom-equipped refueling.

For boom-equipped refueling aircraft, such as the KC-10, KC-135, and KC-46, the receiving aircraft maintains formation based on a relative position to the tanking aircraft (Figure 2). Inside the aircraft providing fuel, a crewmember maneuvers the boom towards a receiver port on the receiver aircraft to complete the engagement.

### 2.2. Conceptual Framework for Air-to-Air Refueling

This section describes the conceptual framework for air-to-air refueling; it is important to have a strong conceptual understanding of the process to understand the sensors required for automation.

To begin air-to-air refueling operations, aircraft must maneuver to achieve a visual formation between the receiver aircraft or section of aircraft and the tanker. Even though the ATP-3.3.4.2(D), the current NATO Standard manual for air-to-air refueling at time of publication, outlines seven rendezvous (RV) procedures, the same general safety procedures apply to all RV [6,17]. The NATO refueling procedures vary depending on factors such as the available communications (e.g., whether ground-based radar is available), the purpose (e.g., combat or not), whether the aircraft are directed by a radar control station (ground based, seaborne or airborne), and the participants (e.g., whether the tanker and aircraft are from the same base). Example procedures include:

*1*. A heading-based procedure that utilizes air-to-air equipment on both tanker and receiver.
*2*. A heading-based procedure that allows an airborne intercept radar to control the procedure upon radar contact.
*3*. A procedure in which the receiving aircraft maintains a specified track and the tanker maintains a reciprocal track at a predetermined offset.

**Figure 3:**
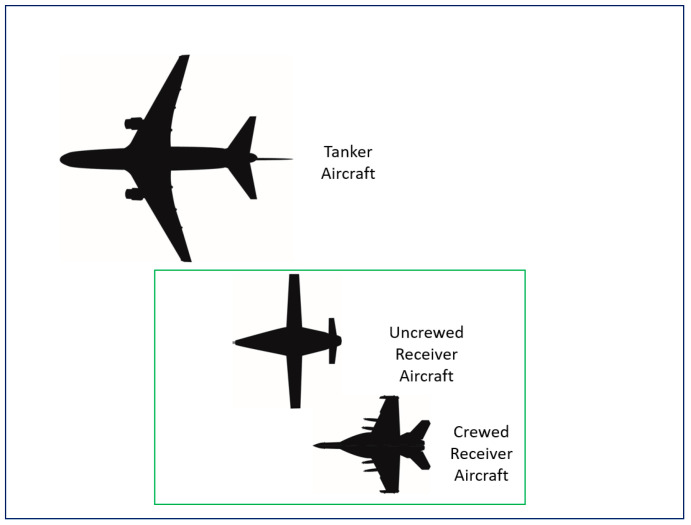
Visual depiction of left echelon formation (tanker at top and two receiving aircraft at bottom).

In all cases, all aircraft, unless specifically directed, use the standard altimeter setting of 29.92 inches, which is verbally confirmed during the initial RV call. While joining, the receiver aircraft should maintain a minimum of 1000 ft. of vertical separation stepped down from the tanker until a visual join can be commenced, usually inside of 1 nautical mile (NM). To control closure, both the tanker and receiver aircraft should fly the speeds prescribed in their respective flight manuals or National Standards Related Document (SRDs). For probe-and-drogue configured tankers, receivers will normally, unless specifically briefed, join in left echelon outside of any aircraft already in position (Figure 3). This allows for sequencing of receivers and monitoring of receivers by the tanker aircrew.

**Figure 4:**
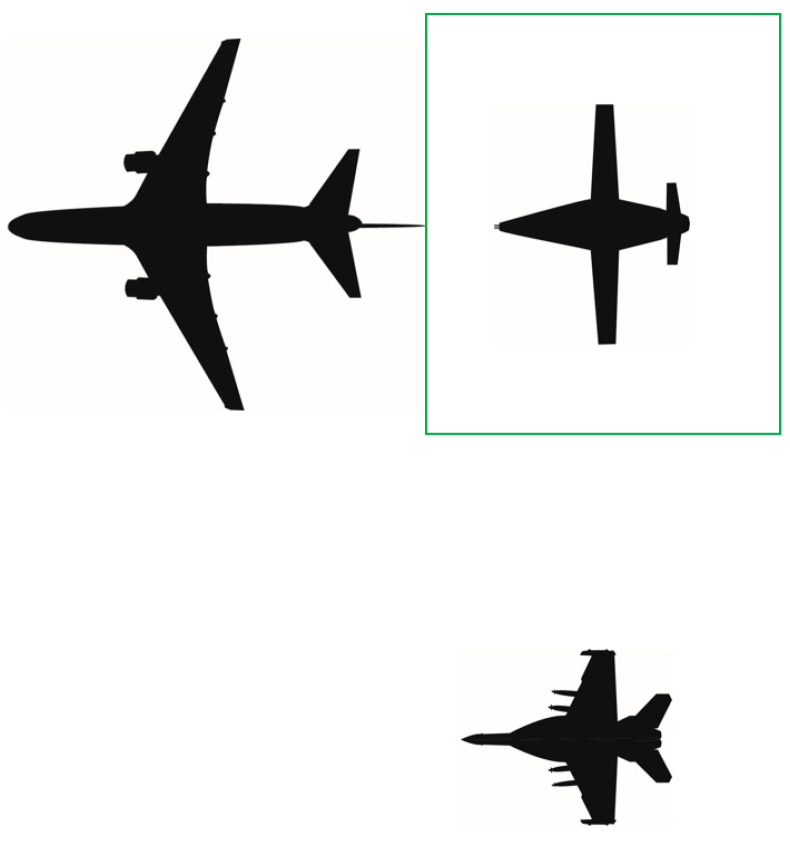
Astern position aft of a centerline drogue (tanker at top left; receiving aircraft at top right).

Once cleared, the receiver aircraft will transition to the astern position located approximately 10 to 15 feet aft of the drogue (Figure 4). Of note, if coordinated prior to the mission or established in Standard Operating Procedures, the receiver may be cleared directly to astern, removing the necessity for the receiver to establish in left echelon.

**Figure 5:**
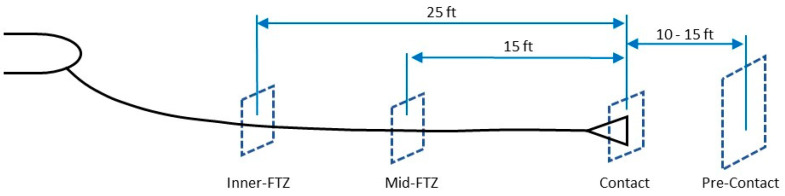
Fuel transfer zone example [18].

Relative to the position of the tanker aircraft’s fuselage or wing, the drogue will nominally flow in the airstream 50 to 90 feet aft of the tanking platform (Figure 5) [18]. Therefore, an astern position can be thought of as a position 60 to 100 feet aft of the wing of the tanker aircraft for the receiver aircraft to prepare for refueling.

**Figure 6:**
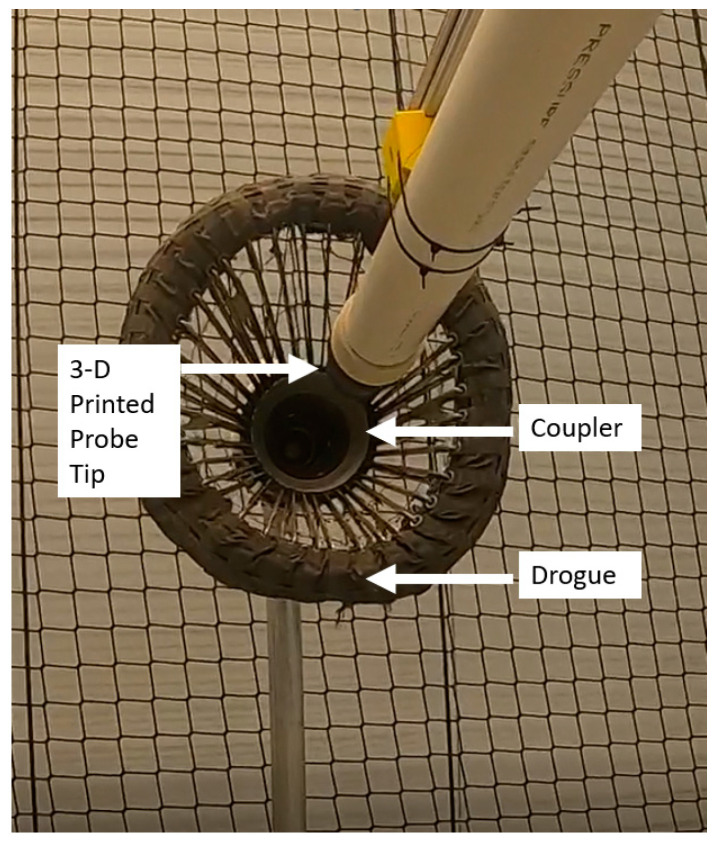
United States Naval Academy probe-and-drogue test equipment.

To conduct probe-and-drogue refueling, the receiver pilot must visually identify the drogue within the domain to maneuver the receiver aircraft’s probe tip into the coupler of the drogue (Figure 6).

During air-to-air refueling, the human pilot must perform object detection and localization within the pilot’s field of view (FOV). After identifying the drogue in the astern position and determining the location relative to the probe tip installed on the receiver aircraft, the pilot must then reduce the separation between the coupler and probe tip by increasing closure. The nominal closure rates range from approximately 1 to 3 knots of closure.

During the approach, the pilot must continually input small flight path adjustments via the control stick to account for the drogue movement in free air. The movement of the drogue can be attributed to multiple factors to include wing flex of the tanker wing, environmental conditions such as turbulence, and finally the creation of a bow wave, which may be attributed to different aspects of the receiver airframe, as it interacts with the drogue during the final moments before engagement [18]. Additionally, the pilot must account for environmental effects on the receiver aircraft and aircrew, such as turbulence, degraded visibility, or challenging lighting scenarios. Depending on environmental conditions, these approaches may become challenging for even the most experienced pilot and may require multiple attempts to safely engage the probe tip and the coupler.

Throughout the approach, the pilot must assess the probability of successful engagement. Outside of the nominal outcome of a successful engagement of the probe tip into the drogue coupler, sub nominal outcomes include “lipping the basket” by contacting the outer ringer of the drogue or the drogue impacting a portion of the receiver aircraft, such as the nose cone or canopy. These conditions may result in damage ranging from minimal to safety of flight depending on the severity of the incident.

If the pilot assesses the engagement will not be successful, the pilot must discontinue the approach by decreasing the closure rates and increasing the separation between the receiver aircraft and the drogue in a safe and controlled manner. The decision to reattempt engagements after a failed attempt will be made after an assessment of the root cause of the failed attempt. Based on any damage during the failed attempt, identification of root cause of the error, probability of error correction, and on mission planning factors to include acceptable airfield availability, allowable level of risk for success of the mission, and physiological state (fatigued, hypoxic, airsickness, etc.) of the pilot, the pilot may reattempt the engagement if deemed necessary.

Once the pilot has successfully engaged the probe tip and the coupler of the drogue, the pilot must continue to decrease the range between the tanking aircraft and receiver aircraft by approximately 5 to 25 feet to enter the Fuel Transfer Zone (FTZ), as annotated on the long hose markings. The FTZ represents a corridor in space determined by the length of hose extended from the tanker aircraft. When the receiving aircraft is in the FTZ, fuel will begin to transfer from the tanker aircraft to the receiver aircraft. When the hose is fully extended prior to engagement, no fuel is being transferred. As the receiver aircraft engages the coupler with the probe tip and begins to decrease the range between the tanking aircraft and receiver, the hose will begin to retract, but fuel will not transfer until the hose reel retracts to a predetermined distance, which represents the FTZ. The pilot receives visual confirmation upon entering the FTZ through a combination of lights installed on either the fuselage of the tanker aircraft or the ARS connected to the wing, as well as the hose markings being in the correct positions. Once established in the FTZ, the pilot then transitions to formation position keeping. Directional position keeping uses the tanker aircraft wing, fuselage, or other marking to help maintain appropriate vertical and horizontal separation. Forward/aft positioning within the FTZ is done using the hose markings on the tanker hose by keeping the first long marking within the tanker hose outlet.

The amount of time required to complete aerial refueling depends on a multitude of variables to include: the tanker’s fuel delivery rate, the maximum fuel rate the receiver can accept, and the total amount of fuel to be transferred. The receiver can confirm fuel flow through the static green light on the drogue system signal light or on their fuel quantity management indications in the receiver cockpit. Two methods exist for determining that the receiver aircraft received the desired amount of fuel. Either the tanker aircraft programs the desired amount of fuel to transfer or the receiver pilot monitors the fuel quantity to determine the desired amount has been transferred.

**Figure 7:**
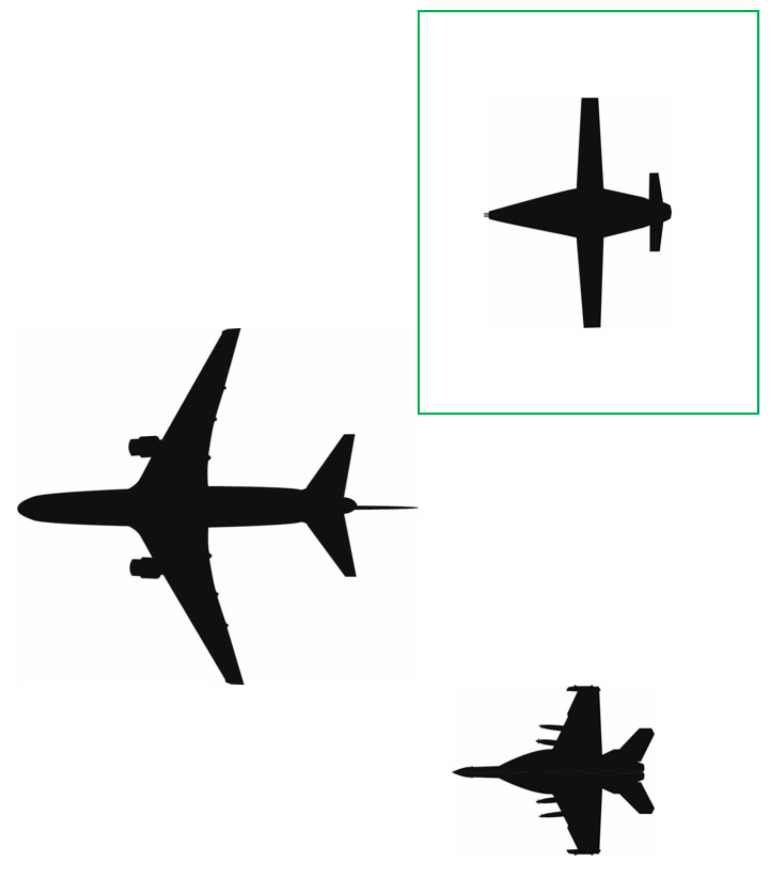
Visual depiction of right echelon formation (tanker at left; refueled aircraft at top right).

Once complete with the transfer of fuel, the receiver will begin separation by decreasing the energy state of the receiver to increase the distance between the tanker and receiver. As the receiver begins increasing the range from the tanker, the hose will begin to unreel to increase the length available until the hose reaches the maximum length attainable. Upon reaching the maximum length of the hose, the probe tip will disengage from the coupler and the receiver aircraft begins to transition to right echelon to either hold until the wingmen are complete or complete final administrative procedures prior to transitioning to the next phase of the mission (Figure 7).

Aerial refueling requires experienced pilots to identify objects in free space, such as the drogue, probe tip, and coupler, prior to maneuvering the receiver aircraft into close proximity of the tanker aircraft to complete an engagement of an object approximately 3 to 4 inches in diameter into a coupler of slight larger proportions. This challenging task can only be completed by adapting the inputs that are dependent on a multitude of environmental factors. While not impossible to automate, a UA capable of A3R would require multiple sensors to provide information to the UA. As of the date of this publication, only demonstrations of this capability have been accomplished.

### 2.3. Innovations for Automated Air-to-Air Refueling (A3R)

While no UA currently exists in the Department of Defense (DoD) that has been authorized to conduct the A3R portion of a mission, the task has been accomplished on both crewed aircraft and UA as a demonstration. Extensive research has been conducted on the algorithms required to complete the task from as a receiver, specifically the computer vision (CV) portion of A3R [19,20,21,22,23,24,25,26,27,28,29,30,31,32,33,34,35,36]. As ranges increase for UAs to travel to complete the mission, the demand for a UA capable of A3R will continue to increase as well.

The first crewed aircraft to demonstrate A3R resulted from a combined research effort between the National Aeronautics and Space Administration (NASA) and the Defense Advanced Research Projects Agency (DARPA) in 2005 and 2006 [37]. Phase 1 of the program involved demonstrating A3R between a crewed F-18 aircraft acting as the receiver and a crewed Omega Air B707 tanker. In this case, the pilot in the receiver aircraft allowed the autonomous agent to control the flight path of the receiver aircraft to complete the engagement. For safety and risk mitigation, the pilot served as a human-on-the-loop (HOTL) and would be able to override the autonomy to safely separate the two aircraft in the event that the approach became unsafe or the autonomous agent began to operate in an unsafe manner. At the completion of the research program, NASA/DARPA successfully designed, developed, and tested a prototype system that was capable of completing the A3R portion of a mission, however this capability was never integrated into a fielded platform.

On 22 April 2015, the USN, using the Northrop Grumman built X-47B Unmanned Carrier Air Vehicle Demonstrator (UCAS-D), demonstrated the A3R portion of the mission using the X-47B as the receiver and an Omega Aerial Refueling Services Boeing 707 tanker [38]. The demonstration program was then discontinued and the X-47B demonstrators were relocated for storage and for public viewing in museums.

Since the completion of the X-47B demonstration program, the USN has not conducted additional A3R testing with a UA acting as the receiver aircraft. In 2021, during the initial testing of the Boeing produced MQ-25, the USN did demonstrate the capability of a UA to serve as the tanker aircraft when a crewed F/A-18F [39], E-2D [40], and F-35C [40]. Flown by Air Test and Evaluation Squadron (VX) VX-23 and VX-20 test pilots, all completed successful engagements with the MQ-25. VX-23 [41] and VX-20 [42] are the Navy’s developmental test squadrons charged with supporting Research, Development, Test and Evaluation of fixed wing aircraft at Naval Air Station (NAS) Patuxent River, Maryland. Given that the USN demonstrated the ability to receive fuel autonomously with the X-47B in 2015 and provide fuel as a tanker autonomously with the MQ-25A in 2021, a logical progression in capability would be to demonstrate a UA aircraft receiving fuel from a UA tanker in the future.

### 2.4. Crewed vs. Uncrewed Probe-and-Drogue Refueling

Aerial refueling has been an integral capability supporting both strategic and tactical aircraft within the DoD for decades [1]. Historically, it has relied heavily on the skills of highly trained human pilots for successful execution [43]. Improvements in both UA and modern sensor, robotic, and control technologies could now enable A3R in UA with an acceptable level of assurance and risk. To complete A3R, the UA must be able to perform all the functions of the pilot beginning at the initial rendezvous between the UA and the tanker aircraft and ending with the UA safely separating from the tanker to continue onto the next portion of the programmed mission. Using the current NATO guidance for A3R, the phases and tasks have been organized into five phases and seven tasks [44]. To enable a UA to complete A3R, at a high level, the UA and tanker must complete the following seven tasks (many of these tasks are supported by pilot skills in conventional aerial refueling):

Phase 1. Transitioning from Execution Portion to A3R Portion of Mission.
  1. Establish communications. Prior to initiation of refueling, communications and datalinks must be established between the UA and the tanker, and any supporting entities that are involved, such as the Ground Control Station (GCS) and Aerial Vehicle Operator (AVO). The GCS receives all pertinent data related to the A3R and the AVO monitors the performance of the UA (the AVO is analogous to a remote safety pilot). As technology advances, the role of the AVO will likely diminish as additional assurance in the system becomes available.
  2. Determine relative position. Determine the initial position of the UA relative to the tanker.
Phase 2. UA Receiver Joins on Tanker.
  3. Decrease separation to astern proximity. Once cleared by the tanker, decrease the separation of the two aircraft in a safe and predictable manner.
Phase 3. UA Transitions from Astern to Engagement.
  4. Transition to computer vision (CV). Once cleared by the tanker, arrive at the astern position to transition from position keeping provided by a data link or navigation aid to position keeping provided by a CV system. Object identification is required to provide location information to the guidance, navigation, and control (GNC) system; this information will then be translated by the UA to decrease the distance between the probe tip and the coupler, once cleared to contact by the tanker.
  5. Position keeping during refueling. Transition back to relative position keeping upon successful engagement to allow fuel to transfer within the FTZ.
Phase 4. UA Receiver Separates from Tanker.
  6. Initiate separation after refueling. Once refueling is complete and cleared by the tanker, the receiver aircraft decreases airspeed to begin to increase the distance between the receiver and tanker. Upon reaching the limits of the hose reel, the probe tip will disconnect from the coupler and the receiver returns to astern position prior to transitioning to right echelon, when cleared by the tanker, for final administrative procedures.
Phase 5. UA Receiver Proceeds on Mission.
  7. Final separation to enable independent flight. Once cleared by the tanker, safely maneuver to increase separation between the two aircraft such that the UA can transition away from relative position keeping to continue the next mission task.

**Figure 8:**
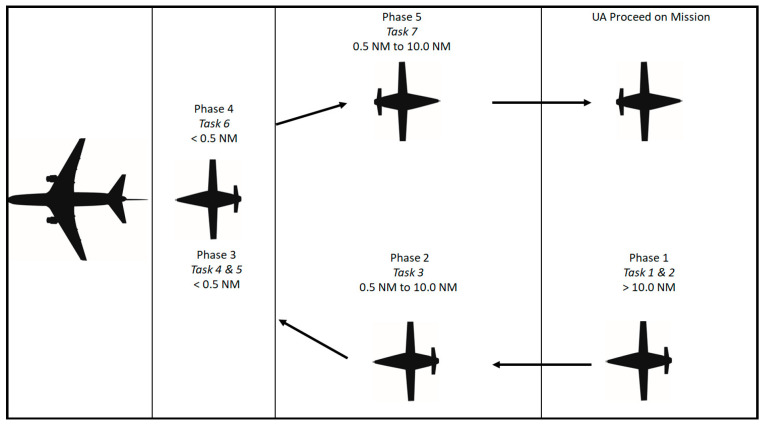
Visual depiction of the A3R tasks by phase.

**Figure 9:**
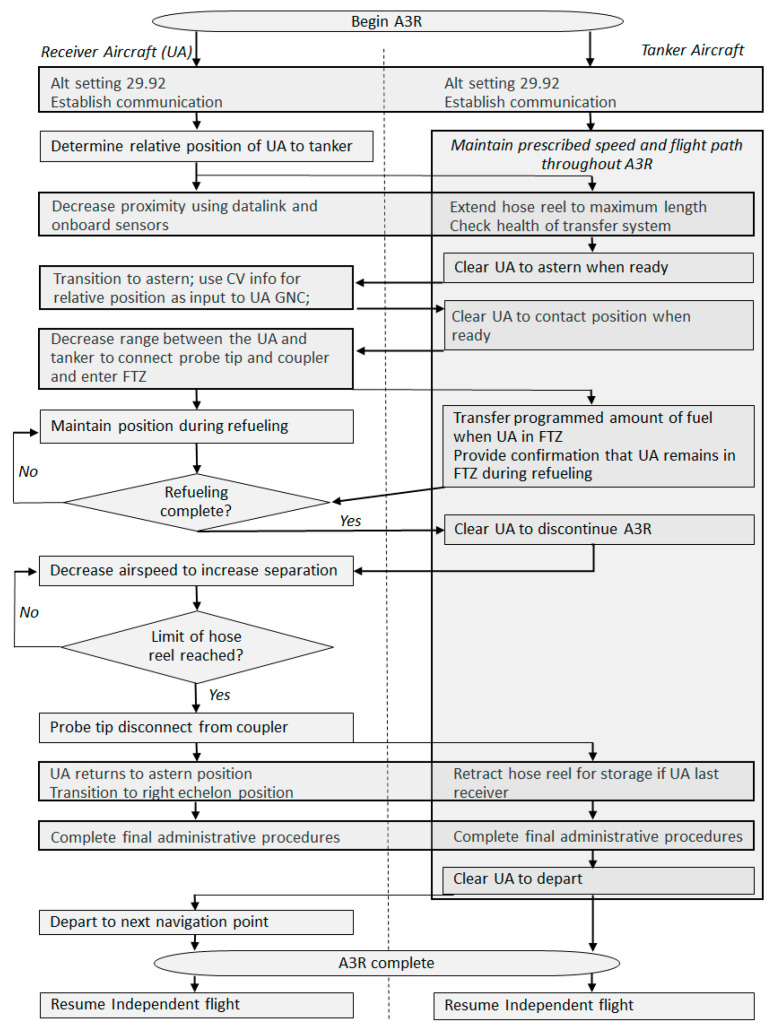
Process map of the A3R tasks.

The relative location of the receiver and tanker begins at maximum separation in the bottom right of Figure 8 during Phase 1, task 1. As the receiver comes the five phases, the receiver moves clockwise through Figure 8 until proceeding onto the next mission task. A process map of A3R activities is shown in Figure 9.

Technology, sensors, and supporting control systems may be located on the UA, on the drogue, on the tanker, or on peripheral sites; for simplicity, this discussion will assume that the technology is on the UA and tanker only. It is worth noting that advances in guidance control systems for other applications, such as landing UAs on moving platforms [45] and landing commercial space vehicles on marine vessels, is likely to advance the guidance and control systems for UA and A3R.

**Figure 10:**
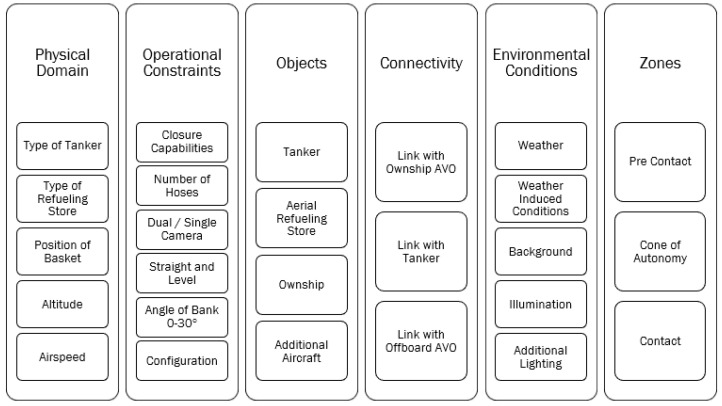
An example of an ODD for a UA tasked with A3R.

The UA must be able to complete all guidance and control tasks throughout the entire operational design domain (ODD) or the domain in which the system is intended to operate. The ODD of the UA will be determined by the desired flight envelope (airspeed, altitude, angle of attack, etc.) capabilities of both aircraft, the need to operate in different environmental conditions (rain, icing, etc.) and the requirements of the intended mission of the platform (daytime only, carrier based only, etc.). The ODD will drive not only the sensor requirements of the platform, but also the assurance requirements of the test program. An example ODD was built from guidance provided by the National Highway Safety and Transportation Administration (NHSTA) [46]; the ODD includes the six categories of physical domain, operational constraints, objects, connectivity, environmental conditions and zones that must be accounted for during the design and certification of a UA tasked with conducting A3R (Figure 10).

In early deployments and during system development, it is common to limit the ODD to evaluate new technologies. For example, most systems are initially evaluated in clear, daylight conditions with unrestricted visibility and limited wind. As systems are matured and refined, operations in different portions of the ODD expand to encompass more challenging environments, such as night or reduced visibility operations, that reflect broader operating conditions. The operational constraints may reflect characteristics of the aircraft, as well as the sensing, control, and communications hardware and software.

Removing the human from the aircraft eliminates system capabilities (e.g., visual confirmation and decision making in unexpected circumstances), but it also removes the constraints in that system. Without a human, the system can operate in harsher conditions, and safety considerations may change significantly when human life in the aircraft is not at risk. Although not listed explicitly in the ODD or the tasks, safety remains a top priority for all aviation activities, including air-to-air refueling. Safety includes the safety of any aircraft occupants, safety of other aircraft in the vicinity, and safety of people on the ground.

## 3. Review of Sensor Requirements for A3R

The previous sections provided a description of A3R and a conceptual framework for the required steps for A3R for a UA. This section provides a discussion of the sensor requirements for UA A3R. These sensors support the tasks listed in Section 2.4, as well as considerations associated with the ODD and considerations related to safety.

### 3.1. Method for Sensor Selection and Limitations

The framework for A3R described in this paper was developed using the current NATO standards for crewed United States Aerial Refueling [6] and uncrewed A3R [44,47]. These UAs must be over 1320 pounds at Maximum Gross Takeoff Weight (MGTW), fly above 18,000 feet (ft.) Mean Sea Level (MSL), and be able to fly at any airspeed. Both the X-47B and MQ-25 mentioned in the history of A3R are Group 5 UAs.

When determining types of sensors required for specific tasks, if a transmit or receive requirement exists, knowing the required frequency band will help determine the size, weight, power, and cost (SWaP-C) [48]. Therefore, when applicable, a frequency band will be provided for each sensor based on previous research with the understanding that new sensors could be designed to satisfy the task while operating in a different frequency band [49]. Research of sensors that a Group 5 UA could not carry due to SWaP-C or due to technology immaturity were not included in this analysis. For many of the tasks, multiple sensors could provide the required information to complete the task, and therefore, the concept of sensor fusion and concerns related to the fusion of information will be presented as well.

Many of the sensors discussed are installed on platforms currently or previously included in the NATO inventory. Due to potential distribution limitations, only open-source articles were included in this analysis of sensors that could complete the required task. While the Lockheed Martin website lists that the F-35, the newest fifth-generation fighter in the NATO inventory, includes an active electronically-scanned array (AESA) radar, distributed aperture system (DAS), and Electro Optical Targeting System (EOTS) [50], information on those specific sensors is limited, and therefore, the concept of including an air-to-air (A/A) radar onboard a UA to provide relative location will be introduced using academic research on the current improvements to X-band radars.

### 3.2. Ensure Communications

To complete A3R, reliable data links that support both voice and data transfer are critical. Data transfer is important for automation functions and monitoring; voice transfer is needed for coordination and communication to support humans that are monitoring and/or interacting with the system. Communications are needed between the tanker and the receiver for automated operations, and the requirements for apertures include both transmission (T) and receiver (R) capabilities.

In addition to communication between the tanker and the UA, there must be communication with the GCS for the AVO to monitor the UA. Communication with the AVO enables a human or team of humans to monitor the operation of the tanker and potentially multiple receivers. Similarly, multiple GCSs controlling multiple UAs must be able to transmit both data and voice for humans to coordinate operations. A GCS serves as the command and control (C2) node for a UA or team of UAs while providing flight information to the AVO in a similar fashion to that of a cockpit instrument display panel in a crewed aircraft [51].

Depending on the level of autonomy onboard the UA, a data link may or may not be required to the AVO. The airworthiness officials certifying the UA at the platform level may not require a human either in (interacting with operations) or on the loop (monitoring operations) to conduct A3R if additional assurance is provided by a certified Run Time Assurance (RTA) [52]. A RTA serves as a monitor to the autonomy under test (AUT), and in the event the AUT encounters a problem or suffers from a degradation, the RTA will revert control back to an automated recovery controller, similar to an Auto Ground Collision Avoidance System (Auto GCAS), to ensure safe operations [53].

**Figure 11:**
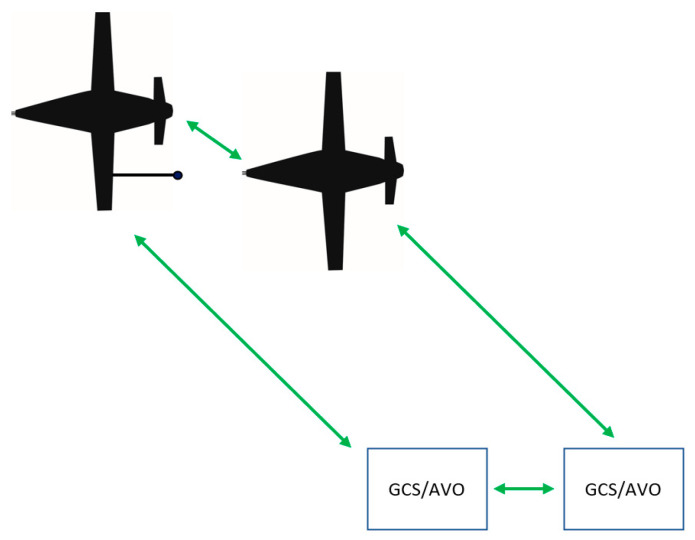
Visual depiction of data links required between multiple UAs and GCSs/AVOs.

A data link to an AVO may be installed for redundancy and/or oversight, but it may not be required for operation. Unless the UA employs a computer vision (CV)-only solution with no fusion of additional location information, such as differential global positioning system (DGPS) data [29], a data link between the receiver and tanker is required for the transmission of position data. For this paper, it is assumed that a data link must exist between both the tanker and receiver, as well as between the AVO and the UAs (Figure 11).

To transmit data and voice between multiple GCSs, if the GCSs are geographically located in a close area, a fiber optic cable or ethernet may be routed to pass information between GCSs [51]. Alternatively, mobile networks operating in ultra-high-frequency (UHF, 300 MHz to 3 GHz), low-frequency (L, 30 to 300 kHz), or S Band (2 to 4 GHz) frequency bands using Commercial Off The Shelf (COTS) components [54] or wireless sensor networks (WSN) in the C frequency Band (4 to 8 GHz, reserved for satellite communications) [55] could be leveraged to pass data and voices between GCSs. If the range between GCSs does not allow for a fiber, ethernet, mobile network, or WSN, either a Line-Of-Sight (LOS) or Beyond LOS (BLOS) solution would be required.

To transmit data between the GCS and UA, either a LOS or BLOS data link will be required depending on the anticipated range between the GCS and UA [56]. Operations within LOS of the GCS allow the UA to have faster and more reliable data transmission. LOS operations are typically limited to operations within the radar horizon, a function of the UA altitude, but also may be further limited due to environmental limitations. For the LOS data link, both the GCS and UA must have a radio installed, such as a software-defined radio (SDR) [57] or a purpose-built radio, capable of producing a LOS data link waveform and an aperture capable of transmitting and receiving the waveform. LOS data links employed by the DoD include the older Link-11 waveform in a high-frequency Band (HF, 2 to 30 MHz) [58], the modern-day Link-16 waveform in the L Band (960 to 1215 MHz) [57], and newer waveforms to include the Multifunction Advanced Data Link (MADL) used on modern day fifth-generation fighters [59]. At the time of publication, no additional information was available about MADL.

In the event the UA achieves a distance where the radar horizon prohibits communication, a BLOS waveform generator and aperture would need to be installed on both the UA and GCS. BLOS communications could occur between multiple aircraft at high altitudes and long distances or between Satellite constellations through Satellite Communication (SATCOM), which operate in the K Band (18 to 27 GHz) [49]. In the event an aircraft cannot receive BLOS information, if the architecture exists, information could be relayed from a BLOS capable aircraft over LOS to an aircraft unable to receive BLOS.

### 3.3. Determine Relative Position

The tanker and receiver aircraft must have an accurate assessment of the relative position throughout refueling. To calculate the relative position, the receiver aircraft could employ a blended solution between the aircraft’s inertial navigation system (INS), Global Navigation Satellite System (GNSS), and the CV system [29]. In 2001, the NASA Dryden Flight Research Center used an instrumentation system called the Formation Flight Instrumentation System (FFIS), built by the University of California, Los Angeles (UCLA). The FFIS fused GPS and INS data and demonstrated relative position estimates down to the centimeter level during a flight test between two F-18 hornets [60]. The A3R demonstrations conducted by DARPA and NASA and the USN also demonstrated the capability of an INS/GPS/CV-blended solution for the A3R mission in 2005 and 2006 [37] and 2015 [38], as mentioned in Section 2.4.

Modern day INS are dead reckoning systems that utilize the initial velocity and position information, as well as information from three-ring laser gyro (RLG) systems mounted on orthogonal axes [61]. After initialization of the system, no additional reference information is required, but the system will drift over time. This drift can be addressed through a multitude of additional sensors, including GNSS information (obtained through a receiver in the aircraft) and signals of opportunity (SOPs).

The ability to integrate GNSS information through a receiver attached to a UA is a critical component of a UA’s guidance, navigation, and control (GNC) system, since it is necessary to address INS drift and since accurate location information is critical for all aspects of uncrewed flights. The space segment of GNSS is comprised of 24 satellites in medium Earth orbit (MEO), the user segment consists of a receiver and ground module, and the control segment for monitoring proper functionality of the constellation. GNSS provides the UA position and time information when in LOS of four or more satellites [61]. Additional GNSS systems have come online recently to compete with GPS and the Global Navigation Satellite System (GLONASS) to include the European Galileo and Chinese Bei Dou [62]. With well over 100 satellites available, once all services reach full operational capability (FOC), UA will benefit from increased Precision Navigation Timing (PNT) capabilities. GNSS signals (L5, L2, L3, and L1) operate in the L Band of the spectrum, and therefore, the UA must be equipped with a aperture capable of receiving the L Band signal [49].

The operational environment is an important consideration when determining the optimal sensors for relative position. GNSS jammers may threaten the ability of an INS/GNSS system onboard the UA [63]. In a contested environment, the UA may only have INS solutions available, but as the UA exits the contested environment and transitions to a more permissive environment, the UA could begin to blend INS with GNSS or SOPs, given the proper radio receivers were installed on the platform, to increase the overall accuracy of the solution. As A3R advances, the tanker and receiver may assess the operational environment and delay execution of refueling until the conditions are more favorable.

Currently in the research phase, SOPs include signals not normally associated with Precision Navigation Timing (PNT) and include AM/FM radio, cellular, digital television, and low Earth orbit (LEO) satellites [64]. UA-mounted receivers’ capability of receiving frequencies associated with those sources (MF, VHF, UHF, K, and L Bands) would be required to refine the spatiotemporal signal landscape while localizing the receiver in both space and time [65].

Having solved for their own ship position, determining the relative position at range comes with the inclusions of different airborne sensors to include A/A TACAN, the A/A radar system, an infrared search and track (IRST) system, or an electro-optical/infrared (EO/IR) system to a UA. As SWaP-C decreases with increases in technology, consideration should be given to adding an A/A radar, an IRST, or an EO/IR system to a UA. Along with providing ranging information to aircraft within the operating area, the target aspect, bearing angle, speed, and closure rates could also be calculated using the same sensors with the assistance of a fusion engine to correlate the information [66]. These sensors would also enable the UA to provide additional information during ISR-T missions while increasing the overall situation awareness available to the AVO. Use of these sensors and all active sensors on the platform will be determined by the emission restrictions associated with the mission, as outlined in previous research [17].

Tactical Air Navigation (TACAN) systems have been in service for decades for both surface navigation and A/A ranging and bear information with reports dating back to 1977 covering the accuracy A/A range and information [67]. Operating in the UHF frequency band, A/A TACAN, also known as “yardstick”, provides a cooperative range and bearing information to a target when systems are set 63 MHz apart. Having the ability to set both X and Y channels, setting 29X on the tanker aircraft would provide a cooperative range and bearing information to a receiver aircraft that has a 92X set. The fusion of land-based TACAN information with INS/GNSS information already exists to aid in airway navigation [68].

Multiple UAs already have a X frequency band radar for Synthetic Aperture Radar mapping in support of ISR-T missions to include Predator B, Global Hawk, and Gray Eagle [66,69]. While previous research focused on using a X-band radar to assist in collision avoidance, this type of technology demonstrates the ability of a UA to fuse radar information to assist in navigation [70]. The ideal transition of technology would be to integrate air-to-air radar information to the navigation solution to arrive within the reception range of DGPS information to continue the join of the receiver on the tanker. As noted in [17], once the receiver aircraft achieves visual of the tanker, the receiver shall cease radiation of the radar by placing the radar into standby.

Additionally known as Infrared Surveillance Systems (IRSS), IRST systems have been employed by military aircraft for decades, such as the system that was installed on the F-14D Super Tomcat. In July 1984, the USN signed a full scale development contract with Grumman to upgrade the F-14A with additional avionics to include the introduction of an IRST system for long-range air-to-air detection [71]. IRST systems work by passively detecting and capturing infrared-specific radiation of target systems, and modern day IRSTs are able to track a target in three dimensions (3D) [72]. Research is already ongoing towards the integration of IRST information with INS/GNSS information installed onboard a UA, with the use case for the analysis being A3R [73].

The last sensor, the EO/IR system, will be discussed to support decreasing the range of the receiver to the tanker to arrive at the astern position. When operating multiple UAs with crossing flight paths, collision avoidance systems become a critical safety component for safe and efficient operations. In terms of perception, EO/IR systems and tracking algorithms have been researched to assist active sensors, such as sonar or radar, in providing perception for a collision avoidance system to ensure safe deconfliction [74,75]. Understanding that these EO/IR systems provide perception for avoidance, these systems could also use the same perception information to inform a GNC on how to join and maintain formation with another aircraft [76]. Additional discussion to follow about using an EO/IR system for drogue tracking to achieve engagement of the probe tip and coupler.

Fusing all the available information into a blended solution will require synchronization among all contributing sensors [29,68,77]. The T&E of sensor fusion engines will require a combination of ground truth data, as well as global and local metrics without ground truth data [78]. An integral problem to solve with sensor fusion will be ensuring the time synchronization and update rates between all contributing sensors have the precision and frequency required to enhance the blended solution and not inadvertently degrade the solution [79]. Without fusion or with a suboptimal fusion solution, the UA will be operating with decreased awareness to the environment, which will be a critical consideration for airworthiness officials to consider when conducting the risk assessment of the UA [80].

### 3.4. Decrease Separation to Astern Proximity

As the separation between the aircraft decreases, the requirement for more precise location and time data increases. This is analogous to the requirement that commercial airliners have an increased need for accuracy as they approach landing: airliner accuracy requirements are 2.0 nautical mile (NM) enroute, decreasing to 1.0 NM for the terminal approach, and further decreasing to 0.3 NM for the final approach [81]. A similar cone of required precision exists as the receiver UA approaches the tanker aircraft.

As the receiver UA decreases the range separation to the tanker, DGPS can be used to support the increased accuracy for the UA. DPGS has a multitude of applications for air navigation [82], including aircraft position [83], reference positioning between vehicles for both air breathing [84] and space-based vehicles [85], and approach capabilities for both shipborne [86] and land base recovery [87]. Modern day DGPS systems have positional accuracy less than 1M and near-nanosecond time accuracy [26]. The SWaP-C associated with installing an aperture on the drogue to obtain GPS information of the drogue is prohibitive though, and therefore, the GPS receiver must be installed on both the receiver UA and the tanker aircraft. To obtain a carrier phase-based solution with regards to the relative position of the receiver UA, the tanker aircraft will need to transmit GPS information to the receiver UA [26]. By transmitting the raw GPS over a data link, such as Link-16, to the UA, a precision relative navigation (P-RELNAV) solution becomes available, which enables faster initialization, a robust operation due to aperture obscuration, and an insensitivity to carrier lock loss [88].

Transitioning from a possible contested environment (for example, an environment in which GPS is denied) to a more permissive environment to conduct A3R, the UA must be able to transmit and receive position information both (1) to the GCS over BLOS or LOS data links and (2) to the tanker aircraft to create a P-RELNAV solution. To complete the P-RELNAV solution, this requires GPS receivers on both the tanker and UA in L-Band [49] and a data link between the tanker and UA, such as Link-16 [57].

### 3.5. Transition to Computer Vision

Once the UA and the tanker are in closer proximity, as defined by the astern position (10 to 15 ft. aft of the drogue), it is necessary to transition to CV to provide guidance information to the GNC for the placement of the probe tip into the drogue coupler. Given the limitations associated with DGPS, demonstrations to date [37,38,89], and academic research on the A3R portion of a mission, a pivot to a CV solution is most appropriate for the final engagement [19,20,21,22,23,24,25,26,27,28,29,30,31,32,33,34,35,36]. As the aircraft moves to an astern position (Figure 4) using an INS/GNSS/DGPS-derived position solution, the camera system installed on the UA can begin to detect objects within the domain.

For the camera, either an EO or IR-based system provides benefits and drawbacks. Research has addressed use of a single monocular camera [32], a 3D Flash Laser Imaging Detection and Ranging (LIDAR) camera [89], common colored industrial cameras [90], stereoscopic cameras [35], and a near-IR spectrum camera [29]. The next section elaborates on these options and provides additional details regarding the context for object identification.

### 3.6. Object Identification to Support Probe Placement

The purpose of a CV system installed on a UA is to detect, classify, and localize objects within the domain. A key aspect of the CV system is that it must be trained on data collected and labeled prior to the system being able to detect, classify, and locate objects within the domain. While organizations have begun to develop frameworks to provide assurance of machine learning algorithms, such as neural networks [91], that could be used for detection, classification, and localization, additional research is still ongoing for completing T&E for a LES [92], such as the CV system that would be used during this portion of A3R.

After detecting objects within the domain, the CV system, trained on data collected during the development of the system, can begin to classify the object detected and provide the information to the GNC. The resulting information is then fused into the UA’s GNC to maneuver the probe tip of the UA into the coupler portion of the drogue attached to the tanking aircraft. In many cases, their capabilities have been tested through modeling [93,94]; scaled physical experiments [95]; and field experiments that utilize robots, ground vehicles, and other equipment to demonstrate the core technologies involved in A3R at a much lower cost.

A variety of methods have been attempted to detect the required objects within the domain, mainly the drogue, coupler, and probe tip. Some of the most common methods include color information, visible markers, or IR markers [96]. The use of markers or beacons to locate the drogue is considered an active vision system (AVS), whereas passive vision systems (PVS) require no additional hardware [22]. The cost to modify and maintain the entire NATO inventory of tanker aircraft to enable AVS would require extensive coordination and commitment from all participating NATO countries. A primary benefit of a PVS is that it requires no additional hardware on the drogue or the tanker aircraft. The primary detractor of a PVS is degraded performance in low visibility or low lighting domains. The limitations associated with low visibility or low lighting conditions may be overcome with the addition of a probe illumination source, such as the lighting system installed on the F-35 family of aircraft [97].

Less commonly, acoustic methods have been suggested for object identification, as well as for air navigation applications [98]. Although not fully developed, acoustic sensors have a strong potential and may offer significant advantages, especially in a multi-sensor navigation system [98]. Although there has been no published research directly related to acoustic methods for the aerial refueling of UA, there has been research published documenting stable and efficient performances for communications for uncrewed underwater vehicles and uncrewed surface vehicles [99].

While transitioning from astern to contact, using DGPS to form a corridor of autonomy (COA) as described in [95]; this COA will bound the position of the UA by allowing for autonomy only when located within the COA. The COA can be thought of as a geofence built from information provided, and the DGPS ensures the safe separation between the tanker and receiver aircraft. If the UA reaches a limit of the COA derived by DGPS, the UA will retrograde to a safer outcome, which could be defined by the center of the COA or may require returning to the astern position.

When considering the camera system installed on the UA, many options exist, depending on the expected domain of operation. Using an EO system coupled with an illumination source capable of illuminating objects within the domain from the astern position represents one of the many different combinations available for satisfying the perception requirement of A3R. Enabling a UA to exercise higher levels of autonomy by utilizing a CV system within the COA enables completion of the positional transition from astern to contact and currently represents the best method available to complete this portion of A3R.

### 3.7. Position Keeping during Refueling

**Figure 12:**
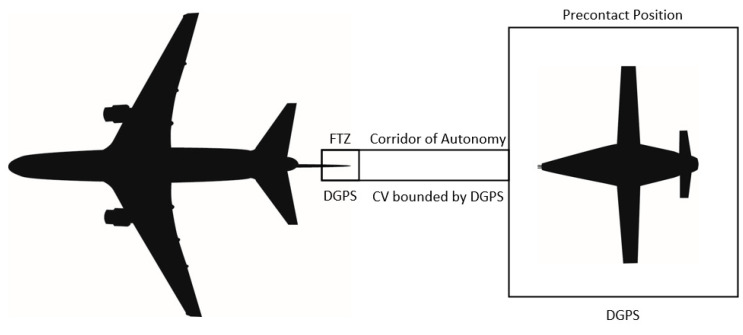
Transition of sensors from Astern to FTZ.

Once the probe tip has connected to the coupler portion of the drogue, the UA must continue to push the drogue towards the fuselage of the tanker aircraft, if the drogue is installed on the centerline, to enter into the FTZ (Figure 5). If the drogue is installed on the wing of the tanker, the UA must push the drogue towards the wing of the tanker aircraft. Since the probe tip and drogue must be included in the camera system Field of View (FOV), when the probe tip connects to the coupler, a large portion of the FOV will be obscured by the drogue. Therefore, once the connection is complete, the UA stabilizes in the FTZ [100], and the air data computer (ADC) faults are isolated to ensure correct inputs are provided to calculate the aircraft state information [101]. DGPS information should be used to create a similar corridor to that of COA for the UA to maintain within until the fuel transfer is complete (Figure 12). This FTZ corridor relies solely on DGPS information blended with INS/GNSS, while CV information is suppressed due to the occlusion within the camera FOV.

### 3.8. Initiate Separation after Refueling

**Figure 13:**
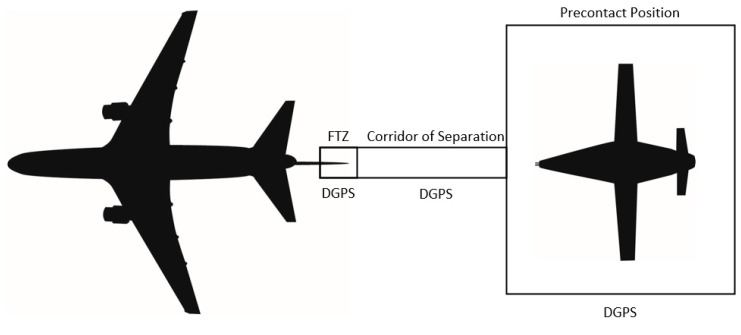
Sensors required for safe separation after refueling.

Once A3R is complete, as measured by the fuel system onboard the UA or the computer that programs fuel transfer on the tanker, the UA must increase separation between the probe tip and the tanker aircraft. This should be accomplished by using DGPS information to maneuver the UA aft of the tanker aircraft to the maximum length of the hose reel attached to the drogue (Figure 13). Note that Figure 13 is similar to Figure 12; however, in Figure 12, the UA is moving toward the tanker, and in Figure 13, the UA is separating from the tanker. At the limits of the hose wheel, the probe tip should disconnect from the coupler, and the UA may return to the astern position. For this portion of the mission, the receiver aperture processing DGPS information is the only additional sensor required.

### 3.9. Final Separation to Enable Independent Flight

Once the UA receives the command for final separation or autonomously decides to separate based on criteria established via preflight planning factors, the UA will execute the same procedure used for joining but in reverse order. Following the separation procedures outlined in [17], the UA will use DGPS to increase separation outside of 10 NM, or a distance determined through mission planning, from the tanker before returning to a blended solution consisting of INS/GNSS navigation using the same sensors previously discussed, but now, the sensors will be used to support increasing separation between the tanker and the receiver UA.

### 3.10. ODD Considerations

The tasks required for A3R are conducted in the context of the ODD presented in Section 2.4. The ODD provides one example of how requirements and test matrices can be methodically constructed. The information in the ODD is largely driven by the concept of employment (CONEMPS), which represents the intended mission and requirements to complete that mission, for the UA. The requirements derived from the ODD may also be used to determine the requirements for sensors installed on the UA.

Consider the physical domain of the ODD. This physical domain includes the type of tankers, refueling stores, position of the basket (wing or fuselage mounted), altitude, and airspeed, which will be driven by the intended tanker and informed by NATO SRDs [17]. The main considerations associated with the sensors onboard the UA will be the ability to complete all phases of A3R within the required domains. Additionally, it will be important to train and verify the CV system to recognize the critical objects for each tanker and refueling store configuration. Anticipated environmental conditions, such as all-weather operations or clear day only, will drive the requirement of an illumination source for the CV or the adoption of a specific camera system.

For the connectivity portion of the ODD, the CV system may necessitate a HOTL for additional assurance, especially in early deployments. Therefore, if the CONEMPS for early operations require a human monitor, a BLOS or LOS data link would be a requirement for the UA to facilitate the required monitoring. Therefore, even if operational deployment would not require a BLOS or LOS data link, early development may require the inclusion of that capability.

Column five of the ODD (Figure 10), environmental conditions, takes into consideration the environmental conditions that a UA may encounter when completing A3R. These environmental conditions will largely be determined by the requirements for the UA to complete the primary mission. If the UA will be required to operate in daytime only under visual flight rules (VFR) weather minimums [2], then the UA will only require sensors that can operate in those weather conditions. In modern militaries, almost all platforms will be cleared to operate in both day and nighttime, as well as most weather conditions. Therefore, the sensors onboard the UA must be able to process information throughout the entire anticipated domain of environmental conditions.

The environmental conditions portion of the ODD will consist of multiple layers to account for all the permutations of conditions the UA may encounter conducting A3R. For example, the weather may include wind, rain, snow, or sleet [46], if the flight envelope of the UA allows for flight through all of these conditions, and these different conditions will have degradation on multiple systems, especially the CV system, as demonstrated by object detection to support autonomous driving [102]. For background, understanding, the types of clouds the UA may encounter will determine the requirements of multiple sensors onboard the UA to include the IRST or A/A Radar [103] or the CV system [104]. In a similar fashion to crewed refueling [97], lighting in the domain, both natural and artificial, must be considered when determining the requirements of the CV system.

The ODD presented provides one example of the factors that need to be defined, the example ODD would be appropriate for a system design and deployment to support the collection of training data and for evaluating the system performance throughout the anticipated domain. The ODD should be designed prior to defining sensor requirements for the UA to ensure that limitations associated with different sensors covered in this paper do not result in unintended limitations of the platform.

### 3.11. Safety Considerations

Safety is a key consideration for all aircraft operations. The sensors onboard the UA support safety directly by ensuring critical functions and indirectly by providing redundancy to operational and monitoring capabilities. For example, the COA defined by DGPS information ensures that, while the UA is in the COA, the UA can exercise a higher level of autonomy by navigating based on information provided by the CV system. The COA also helps ensure that the UA does not inadvertently navigate towards an incorrectly identified object. By confining the approach to a DGPS defined corridor, additional assurance can be provided to airworthiness officials that the UA will operate in a safe manner when a human cannot monitor operation in real time.

Depending on the criticality of the system, additional redundancy may be required for sensors onboard the UA. For example, if the mission of the UA requires a combat radius that exceeds the unrefueled range of the platform, A3R becomes a safety critical function to the success of the flight. For a mission that requires A3R to succeed, redundancy may be required for specific sensors, such as the camera system, to ensure that a single failure does not eliminate the capability for the platform to receive fuel airborne. This requirement will be derived from the CONEMPS and will inform requirements for redundancy throughout the system.

In training, crewed refueling is typically conducted in special use airspace (SAU), as defined by the FAA, to include restricted areas, warning areas, or military operations areas (MOA) containing a published tanker track [2]. Similarly, T&E of new capabilities and platforms will generally occur in the same SAUs, due to the limited presence of other aircraft not involved in T&E. Once a UA capable of A3R has been certified and delivered to warfighters, the UA would then be capable of conducting A3R wherever the mission planning factors allowed for operations. Key considerations here would be deconfliction from other flight operations around friendly forces and the avoidance of threat engagement ranges due to the vulnerability of both platforms during A3R and the strategic importance of tanker aircraft.

The extended flight time enabled by aerial refueling may introduce additional safety considerations for UAs. Previous research has explored the use of sensors and measurements for UA safety and advocates for standard testing to ensure reliability and assess performances [105].

## 4. Discussion and Conclusions

### 4.1. Challenges to Fielding a UA Capable of A3R

While SWaP-C decreases for many of the sensors discussed in this paper and the capabilities of CV systems continue to increase, the possibility of a UA conducting A3R in the future is not a question of if but when. The current DoD acquisition lifecycle consisting of requirements, contracts, development, test and evaluation (T&E), deployment, and sustainment is not optimized to expeditiously acquire a LES, such as the CV system required for a UA to complete A3R. In 2022, the Department of Air Force/Massachusetts Institute of Technology (DAF/MIT) AI Accelerator program published a guidebook to AI Acquisitions, which represented one of the first DoD guidebooks to address many of the concerns related to acquiring a LES [106].

Specifically addressing the T&E portion of the acquisition lifecycle, many challenges exist for adequately evaluating the performance of a UA conducting A3R to include the complexity of the system; safety; security; and lack of methods, tools, and infrastructure, policy, standards, or metrics [107]. In 2020, the USAF published their first guidance to address many of these issues [53], but additional research will be required to create manuals for all services within the DoD.

Luckily, different government organizations, such as the European Union Aviation Safety Agency (EASA), have begun publishing concepts for assuring neural networks, which could be used to provide perception to a UA [91]. Additionally, civilian standard organizations that include the American Society for Testing and Materials (ASTM) [52] and Society of Automotive Engineers (SAE) International [108] have begun to address many of the same concerns the DoD has identified as well.

While, from a sensor standpoint, a UA capable of A3R could very well happen in the near future, extensive research must be completed, and challenges addressed prior to the successful fielding of a UA capable of A3R. Given the severity to mission success of a UA failing to complete A3R, resources must be committed immediately to begin to address the challenges noted above and to ensure a successful acquisition.

### 4.2. Conclusions

**Figure 14:**
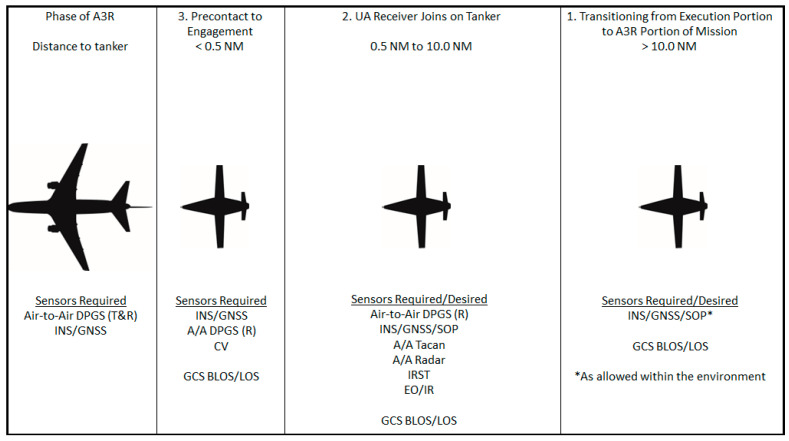
Review of the sensor requirements by phase of flight.

While demonstrated multiple times by DARPA/NASA and the USN, a UA that has achieved Initial Operational Capability (IOC), capable of conducting A3R, does not exist as of time of this publication. Extensive research has been conducted on different aspects of the missions, such as DGPS for close formation flying and CV for object detection within the domain. As technologies advance, new sensors bring additional capabilities that were not available during previous demonstrations. In this paper, a discussion of the sensors required to complete the different phases of A3R was covered, and they are shown in Figure 14. These sensors, and a summary of their purpose, the phase in which they are used, and the frequency band they utilize, are also summarized in Table 1 below. The phases listed in Table 1 correlate with the phases shown in Figure 14.

As discussed in Section 3, the sensor requirements (e.g., transmit and receive (T&R) capability or receive (R) capability) and the anticipated frequency band of the sensor will provide valuable insight into the SWaP-C required to implement that capability. Many of the sensors listed in Table 1 overlap in frequency band, such as the airborne data link between UA to UA and the GNSS receiver. In these cases, resource simulations will be required to determine if the receiver system (aperture, radio, etc.) has the required resources to manage multiple requirements.

In conclusion, all sensors required to complete A3R are available as of the date of this publication. There are numerous benefits from A3R, and the future of military and commercial aviation will necessitate that UAs become capable of completing A3R. This paper introduces and reviews the sensors required for a UA to complete A3R. Suggested future research to support A3R includes the deployment of scaled models, demonstrations of the application of sensors to accomplish the tasks and phases required for A3R, and the investigation of new sensor technologies to support A3R requirements.

## Figures and Tables

**Table 1:** Summary of the sensors covered.

Sensor *	Purpose	Phase **	Frequency Band
Data link: GCS to GCS	Transmit data and voice	1–5	Fiber, Ethernet, UHF, L, & C
Data link: GCS to UA	Transmit data and voice		L & C
Data link: UA to UA	Transmit data		L & Ku
Tanker and Receiver INS	Position and Timing		---
GNSS Receiver	Update INS	1–5	L
SOP Receiver		1, 2	MF, VHF, UHF, K and L
A/A Tacan	Target (tanker) information	1–5	UHF
A/A Radar		1, 2, 4, & 5 **	X
IRST System		1–5	Passive
EO/IR System		1–5	Visual & IR
DGPS Tanker (T&R) *** Receiver (R)	Guidance information	2–5	L & Ku
EO/IR System	CV for guidance	3	Visual * IR

* As allowed within the environment. ** These phases correlate with the phases shown in Figure 14. *** Transmit and Receive (T&R) and Receive (R) sensors.

## Data Availability

Not applicable.
